# Alzheimer mimicry: LATE and PART

**DOI:** 10.1007/s00702-025-02916-0

**Published:** 2025-03-31

**Authors:** Nenad Bogdanovic, Una Smailovic, Vesna Jelic

**Affiliations:** 1 Clinic for Cognitive Disorders M52, Karolinska University Hospital-Huddinge, Theme Aging, 14186 Stockholm, Sweden; 2 Department of Clinical Neurophysiology, Karolinska University Hospital, Stockholm, Sweden; 3 Division of Clinical Geriatrics, Department of Neurobiology, Care Sciences and Society, Center for Alzheimer Research, Karolinska Institutet, Huddinge, Sweden

**Keywords:** SNAP, LATE, PART, Atypical Alzheimer, Dementia, Elderly

## Abstract

Alzheimer’s disease (AD) is the main cause of dementia and accounts for 60% of dementia syndromes in people older than 75 years. The correct classification of AD and non-AD cases is mandatory to study disease mechanisms or new treatment possibilities. A typical clinical picture of AD consists of a progressive cognitive decline, with primary memory impairment. Structural, functional, and molecular brain imaging, along with CSF biomarkers of amyloid pathology, neurodegeneration, and the presence of a vulnerability-associated APOE genotype, support the diagnosis of AD. Use of biomarkers have led to the identification of individuals with mild cognitive impairment who are amyloid-negative addressing a conceptually separate clinical entity named suspected non-Alzheimer disease pathophysiology (SNAP). Clinical presentation and progression of SNAP can mimic AD which makes the final diagnosis and possible treatment uncertain in up to 30% of cases in clinical centers that are not using biomarkers. These non-AD pathologies are common with advancing age both in cognitively impaired and clinically normal elderly people and include Argyrophilic Grain Disease (ARG), Tangle Predominant Dementia and TDP-43 proteinopathy. The terms Primary age-related tauopathy (PART) and Limbic-dominant TDP-43 age-related encephalopathy (LATE) have been proposed as the most common and useful biological and emerging clinical construct to describe this phenomenon in > 80 years old individuals. Current evidence underlines the limitations of existing diagnostic tools, which remain inadequate for fully capturing the complexities of these conditions. Addressing these diagnostic ambiguities is crucial for assigning accurate diagnoses, reducing frequent misdiagnoses of AD, and implementing appropriate therapeutic strategies for elderly patients with mild cognitive impairment and dementia.

## Introduction

**Fig. 1:**
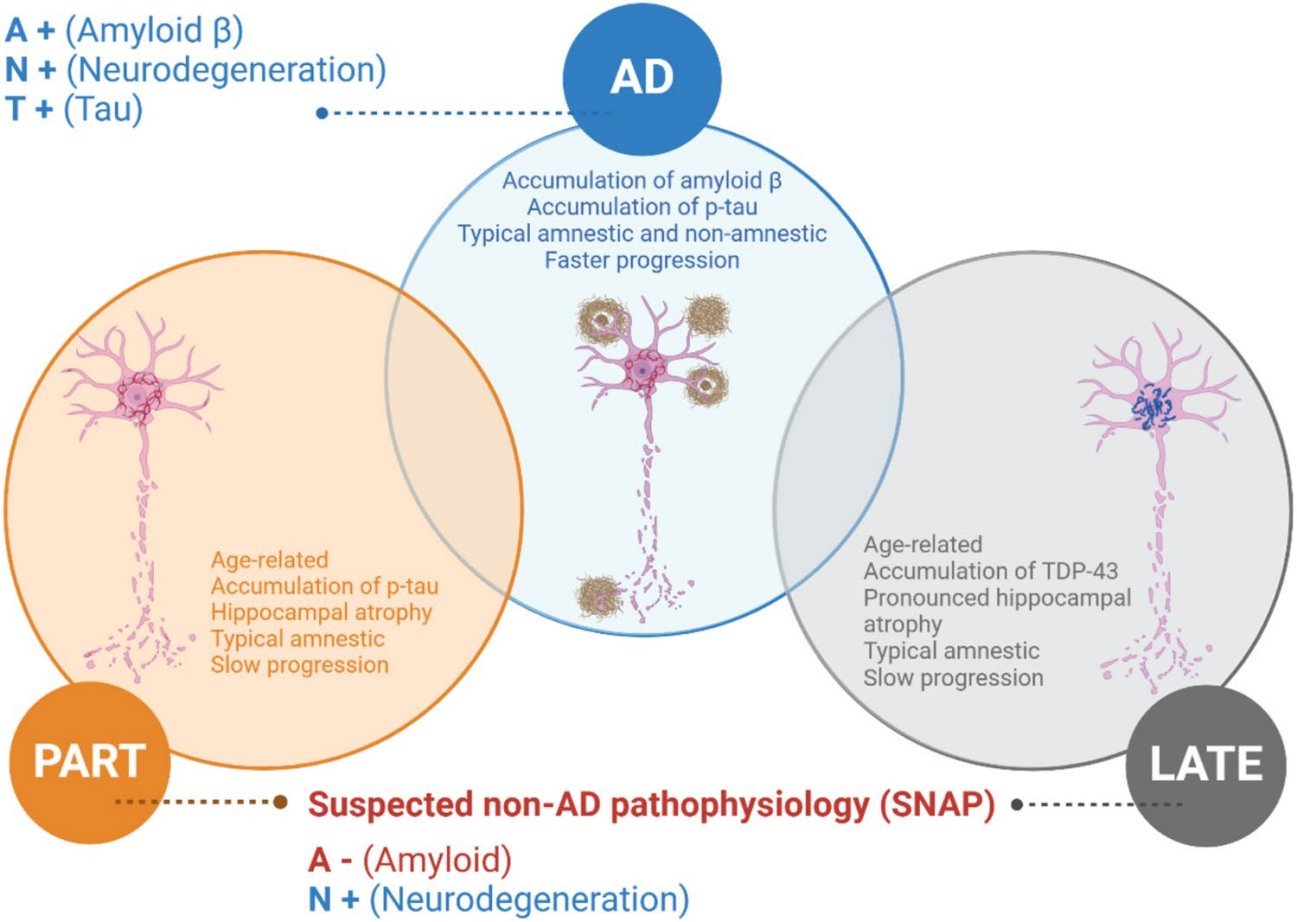
Overview of basic differences between ADNC, PART-NC and LATE-NC. AD = Alzheimer’s disease, LATE-NC = Limbic-predominant age-related TDP-43 encephalopathy neuropathologic change; PART-NC = primary age-related tauopathy neuropathologic change

The most common cause of amnestic syndrome leading to dementia in the elderly is Alzheimer's disease (AD) that accounts for 60% of dementia syndromes in people older than 75 years (Jellinger 2006). Core neuropathological features of AD include presence of amyloid plaques and neurofibrillary tangles (NFTs) (Braak and Braak 1991). However, everyday clinical practice proves that elderly patients can have cognitive impairment and dementia without having amyloid deposits in the brain. For practical reasons, this patient group is called SNAP (suspected non-Alzheimer disease pathophysiology), a construct introduced by Clifford Jack in 2014 when operational NIA–AA criteria for preclinical Alzheimer's disease were formulated (Jack et al. 2014). However, this general and somewhat vague term should be avoided in clinical routine. Neuropathological examination showed that with increased life expectancy, cognitive impairment/dementia can be characterised by the presence of limbic-predominant age-related TDP-43 encephalopathy (LATE-NC) or by primary age-related tauopathy (PART-NC) (Nelson et al. 2019; Crary et al. 2014) (Fig. 1). However, the prevalence and coexistence of these brain pathologies, characterized by high morbidity and distinct clinical features, remain uncertain.

**Fig. 2:**
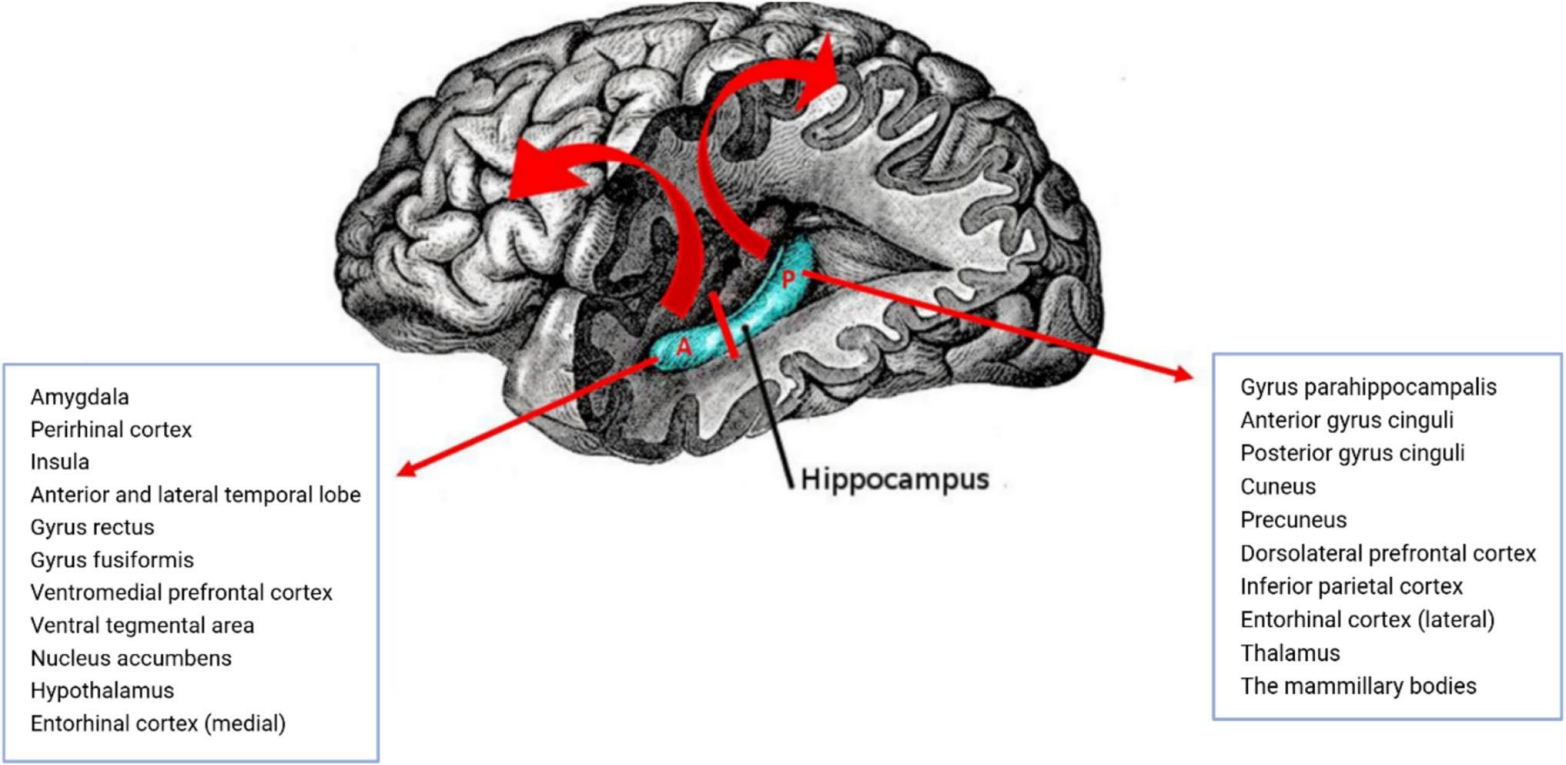
The human hippocampus has anatomically distinct anterior and posterior segments that are associated with different functional specializations, morphology, and connectivity patterns. The anterior and posterior hippocampal projection areas are shown in the left (A = anterior) and right (P = posterior) blue squares

Commonly, these predominantly amnestic syndromes of cognitive impairment and dementia involve limbic structures at the disease onset. The role of the hippocampus, thalamic nuclei, and mammillary bodies in providing the necessary neural substrate for acquiring and retaining new information is well known (Squire 1992). The most extensively studied limbic region is the hippocampus, which has been at the centre of attention since 1956 due to the famous case of H.M., a patient who showed severe anterograde amnesia after bilateral amygdalo-hippocampectomy for seizure control (Scoville and Milner 1957). To understand the pathoanatomical and clinical characteristics of LATE-NC and PART-NC, one must reflect upon the entorhinal cortex/hippocampal anatomy (Hyman et al. 1986). The human hippocampus has anatomically distinct anterior and posterior segments associated with different functional specialisations, morphology, and connectivity patterns (Poppenk et al. 2013) (Fig. 2). Thus, the accumulation of neuropathology in different parts of the hippocampus can lead to distinct clinical presentations. LATE-NC and PART-NC are characterized by accumulation of pathology predominantly in the anterior part of the hippocampus, amygdala, and entorhinal cortex, indicating dysfunction in the anterior hippocampal projection areas (Nelson et al. 2019). In advanced stages, the neuropathology spreads throughout the hippocampus toward the rest of the limbic areas.

### LATE-NC

LATE-NC is neuropathologically characterized by accumulations of transactive DNA-binding protein 43 kDa (TDP-43) (Nelson et al. 2019). This condition has been detected in over 40% of brain specimens in community autopsy series, including participants with an average age of death of 88 years (Nelson et al. 2022). TDP-43 pathology was first discovered in 2006 as a primary pathological feature of frontotemporal lobar degeneration with TDP-43 inclusions (FTLD-TDP), amyotrophic lateral sclerosis (ALS), and later in hippocampal sclerosis with amnestic cognitive impairment not associated with FTLD/ALS (Amador-Ortiz et al. 2007). Additionally, TDP-43 pathology is now considered to occur in many other conditions, such as chronic traumatic encephalopathy and Huntington’s disease (Chornenkyy et al. 2019). Although diagnostic ambiguities still exist in TDP-43 neuropathological assessments, LATE-NC has distinctive features, including the neuroanatomical distribution of TDP-43 pathology, clinical presentation, genetic risk factors, and epidemiology (Coyle-Gilchrist et al. 2016; Crary et al. 2014).

#### LATE-NC vs ADNC

It is important to mention some of the shared features between LATE-NC and ADNC (Table 1). First, LATE-NC is strongly associated with amnestic syndrome of dementia, independently of other concurrent brain pathologies (Nelson et al. 2019). Furthermore, ADNC and LATE-NC are genetically pleiotropic: gene variants associated with ADNC, such as APOE genotype with a vulnerability allele e4, are also associated with an increased risk of LATE-NC (Dugan et al. 2021; Yang et al. 2018). Interestingly, it has been shown that TDP-43 pathology may co-localize with tau neurofibrillary tangles in neurons, suggesting common upstream pathophysiological pathways or synergistic exacerbation of protein misfolding (Smith et al. 2017).

**Table 1:** Overview of neuropathological, epidemiological, genetic, clinical and biomarker differences between LATE-NC, ADNC and ADNC + LATE-NC

	LATE-NC	ADNC	ADNC + LATE-NC
Protein aggregation	TDP-43	β-amyloid, tau	TDP-43, β-amyloid, tau
Prevalence	~ 20%	~ 40%	25–60% of AD patients
Anatomy of neurodegeneration	Hippocampus and amygdala	Hippocampus and amygdala	As in LATE-NC and AD with the difference in Nb Meynert
		Inferior temporal lobe	
	Top of the temporal lobe	Lateral parietal lobe	
	Medial frontal area	Precuneus	
	Orbital cortex	Cingulate gyrus	
	Sparred nucleus basalis Meynert	Nb Meynert	
Genetic risk	TMEM106B, RBFOX1, APOE, GRN	APOE, TREM2, CLU, B1N1	As in LATE-NC or AD
Clinical features	Slow progression of memory impairment	Amnestic and multidomain cognitive impairment	Severe cognitive impairment/ dementia
	Possible behavioural changes		Rapid functional decline
Imaging biomarkers	MRI, [^18^F]FDG-PET	MRI, [^18^F]FDG-PET, amyloid- and tau-PET	MRI, [^18^F]FDG-PET, amyloid- and tau-PET

Even though ADNC and LATE-NC have primarily distinct pathological features, up to 75% of individuals with LATE-NC may have some level of co-morbid ADNC (Nelson et al. 2022). However, large multicentre community- and population-based study showed that more than 60% of LATE-NC lacked severe ADNC (corresponding to Braak NFT stages 0–IV) (Nelson et al. 2022). It has been suggested that TDP-43 proteinopathy in the context of ADNC may be merely an “added” pathology, analogous to the occurrence of Lewy body pathology in the amygdala (Josephs 2019).

**Fig. 3:**
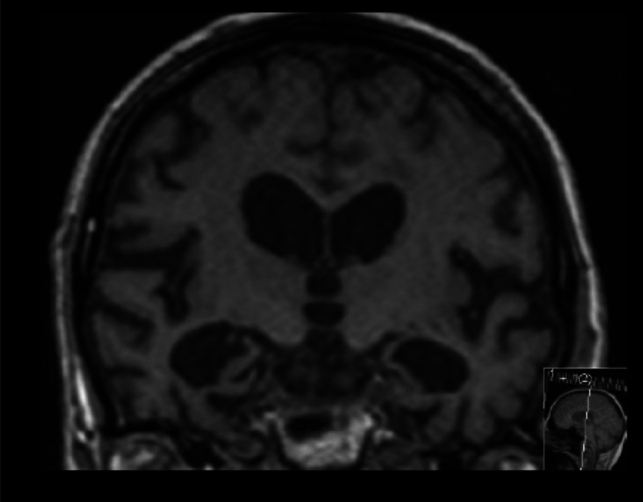
An 86-year-old patient presents with an MMSE score of 25/30 and APOE ε3/3. Cerebrospinal fluid analysis reveals no amyloid or tau pathology. MRI shows severe atrophy in both hippocampi, graded as MTA stage 4*, while cortical atrophy is minimal and notably disproportionate to the hippocampal changes. This presentation strongly resembles the clinical and pathological features associated with “pure” LATE-NC. *MTA (medial temporal lobe atrophy) visually rated by a semiquantitative scale (Scheltens et al. 1992) ranging from 0 to 4, where 4 stand for end stage hippocampal atrophy

The presence of LATE-NC in cases with concomitant ADNC is nevertheless important to note, as ADNC with LATE-NC has a more severe clinical phenotype than ADNC without LATE-NC (Nelson et al. 2010; Robinson et al. 2013). ‘Pure’ LATE-NC is, on average, associated with a less severe clinical phenotype than ‘pure’ ADNC. It is important to emphasize that the clinical presentation of LATE-NC is limited to reduced episodic memory, which is specifically related to the anatomy and the connectivity of the anterior hippocampus (Wilson et al. 2019). The clinical symptomatology of LATE-NC is strongly modified by the extent of ADNC co-morbidity and possibly by other concomitant pathologies, such as hippocampal sclerosis and arteriolosclerosis (Wilson et al. 2019). Recent imaging studies also suggest that brains with ADNC and co-morbid LATE-NC have more pronounced hippocampal atrophy than those with ADNC alone (Dawe et al. 2011; Josephs et al. 2017a; Sahoo et al. 2018; Bejanin et al. 2019; Nelson et al. 2019) (Fig. 3). Additionally, patients with ADNC who have co-morbid LATE-NC are, on average, older (> 80 years), and have a higher ADNC burden, poorer cognitive performance, and a higher tendency to manifest behavioural and neuropsychiatric symptoms compared to ‘pure’ ADNC. Therefore, confirming the presence of ‘mixed’ pathologies is important since the clinical manifestations vary with different combinations of pathologies (Nelson et al. 2024). The comparison between LATE-NC, ADNC and ADNC + LATE-NC is presented in Table 1.

#### LATE-NC clinical features

Individuals with LATE typically report fewer memory complaints and could be cognitively only slightly impaired on standard neuropsychological assessment compared to those with AD or LATE + AD, despite more advanced atrophy in the limbic area. These individuals tend to live longer with their cognitive symptoms emerging later in life and progressing at a slower pace. Cognitive testing showed that LATE patients performed better in various cognitive domains (memory, processing speed) compared to those with AD or LATE + AD. However, comorbid LATE + AD resulted in more severe impairments across all domains (Butler-Pagnotti et al. 2023). Behavioural symptoms linked to LATE remain inconsistent. Some evidence suggests that pure LATE-NC may slightly raise the risk of frontal lobe-associated behavioural symptoms, while pure AD is more closely associated with increased risk for agitation (Liu 2020). Interestingly, the association between hippocampal sclerosis (HS) and LATE-NC does not appear to elevate the risk of epileptic seizures. HS associated with LATE-NC is in general both pathologically and clinically distinguished from HS associated with seizures (Gauthreaux et al. 2022).

A recent proposal introduces the concept of LANS (limbic-predominant amnestic neurodegenerative syndrome), which may indicate the presence of LATE, though it is not exclusive to it (Corriveau-Lecavalier et al. 2024). LANS is defined by a gradual, progressive cognitive decline lasting at least 2 years, primarily affecting memory, with no identifiable alternative causes. The diagnosis also considers optional supportive criteria, which include clinical indicators such as an age of 75 or older, relatively preserved neocortical functions, and early-stage impairment of semantic memory. Structural and functional imaging findings, including MRI showing disproportionate hippocampal atrophy and [^18^F]FDG-PET revealing limbic system hypometabolism with preservation of the precuneus area, thus without the characteristic AD pattern, can further support the diagnosis (Wisse et al. 2015). A low likelihood of significant neocortical tau pathology, as indicated by biomarkers in CSF or Amyloid PET scans, further enhances diagnostic confidence. Common APOE ε isoforms in LANS are predominantly allele ε2 and ε3, with a much less frequent APOE ε4 allele. The certainty of a LANS diagnosis increases with the number of these criteria met alongside the essential feature of amnestic-predominant cognitive profile.

#### LATE-NC vs FTLD-TDP-43

TDP-43 is an aberrant protein aggregate initially discovered in patients with ALS and frontotemporal lobar degeneration (FTLD-TDP), before LATE-NC was distinguished as a separate entity (Neumann et al. 2006). To date, the clear-cut clinical and pathological boundaries between LATE-NC and FTLD-TDP have not yet been fully delineated (Nelson et al. 2019). LATE-NC shares histopathological features with FTLD-TDP type A (Aoki et al. 2015). Furthermore, both LATE-NC and FTLD-TDP present with neuroimaging evidence of frontal and temporal atrophy, even though the degree of atrophy is usually less severe in LATE-NC cases. The main differences between FTLD-TDP and LATE-NC are in epidemiology and clinical presentation. LATE-NC is much more common, affects older individuals, and typically presents as an amnestic cognitive disorder (Nelson et al. 2011, 2019).

### PART-NC

Before the term “primary age-related tauopathy” (PART) was proposed in 2014, pathologists had observed localized neurofibrillary degeneration that was mostly confined to medial temporal regions in the brains of aged subjects with relatively well-preserved cognitive function. These findings were somewhat informally described as “ageing changes” because the characteristics were considered insufficient for a diagnosis of Alzheimer’s disease. The newly proposed consensus term for PART includes neuropathological features ranging from the presence of isolated neurofibrillary tangles in cognitively normal aged brains to tangle-predominant senile dementia (TPSD) and argyrophilic grain disease (AGD) (Crary et al. 2014; Irwin et al. 2016).

Thus, researchers have proposed the consensus term PART-NC as a more objective and quantitative description of pathological disease status separate from the clinical presentation. The term PART-NC is inspired by the pathological classification system of the National Institute on Aging-Alzheimer’s Association for AD (Hyman et al. 2012). Since its introduction, the clinicopathological features of PART-NC have been elucidated in more detail (Bennett et al. 2017; Crary et al. 2016; Josephs et al. 2017b; Kaufman et al. 2018; Neltner et al. 2016).

**Fig. 4:**
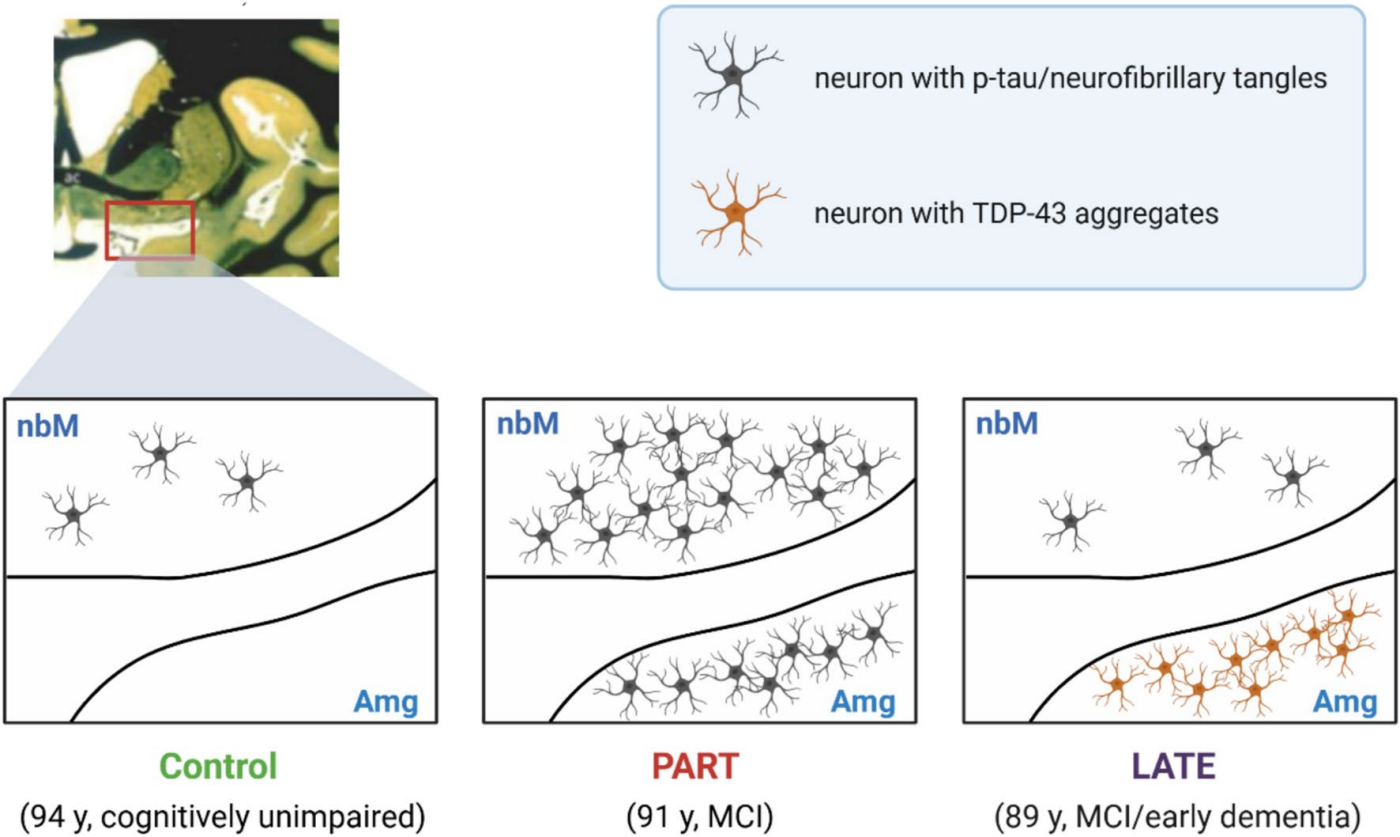
The nucleus basalis of Meynert (nbM), a key cholinergic nucleus, exhibits distinct patterns of involvement in LATE and PART. In LATE, TDP-43 pathology does not affect the nbM, whereas in PART, the nbM is notably impacted by phospho-tau pathology. On the other hand, TDP-43 pathology is commonly found in the amygdala in LATE. In cognitively unimpaired older individuals, minimal tau pathology can often be observed in the brain, which may represent a preclinical or pre-PART stage

The gross features of a brain with PART-NC include advanced atrophy primarily located in the medial temporal lobe, often dominant in the left hippocampus (Josephs et al. 2017b), with or without the presence of very mild and diffuse neocortical atrophy and without or with very few amyloid plaques (Kovacs et al. 2015). In TPSD, AD-type NFTs, including ghost tangles, are mainly distributed in the hippocampus and medial temporal lobe, corresponding to Braak stages I–III in the majority of patients and to stage IV in a few rare cases (Braak et al. 2011). NFTs can also be observed in subcortical structures, such as the amygdala, nucleus basalis of Meynert (Fig. 4), nucleus accumbens, hypothalamus, thalamus, and the olfactory system (bulb and cortex), and in the brainstem, including the substantia nigra, locus coeruleus, dorsal raphe nucleus, and medulla oblongata (Braak et al. 2011; Hickmann et al. 2021).

#### Is PART-NC a Part of the AD Continuum?

Considering PART-NC as a part of the AD continuum, particularly the limbic-dominant form of AD, has been discouraged for several reasons (Josephs et al. 2017b; Kovacs et al. 2015). Firstly, PART-NC is associated with lower Braak NFT stages and fewer, or absence of, Aβ plaques. In the absence of Aβ, as seen in definite PART-NC, tau-positive NFTs or argyrophilic grains tend to be more abundant with older age at death (Josephs et al. 2017b). To what extent presence of amyloid pathology in the form of amyloid and neuritic plaques may still be included in the diagnosis of definite PART-NC requires further studies, however a quantitative threshold for Aβ deposition, up to Thal phase 2, has been suggested for the diagnosis of “possible PART-NC” (Crary et al. 2014). Secondly, patients with PART-NC have a higher age of onset, longer disease duration, and less severe cognitive impairment (Josephs et al. 2015). Thirdly, the frequency of APOE ε4 is much lower in PART-NC than in the normal elderly population (Josephs et al. 2015).

The similarity between tau pathology in PART-NC and ADNC, along with the difficulty in defining when PART (if amyloid pathology is also present) should be classified as AD rather than PART + AD, raises questions about whether PART should be considered a distinct clinicopathological entity (Irwin et al. 2016; Duyckaerts et al. 2015). It has been suggested that a neuropathological diagnosis of PART-NC should be applied conservatively to cases where NFT or argyrophilic grains primarily affect the hippocampus/limbic area, and where there is a lack of Aβ deposits, and no evidence of any other dementia characterized by NFT (Yamada et al. 2003). Finally, a comprehensive neuropathological analysis is essential to gain a deeper understanding of age-related neurodegenerative changes, including the clinicopathological characteristics of PART.

#### PART-NC: clinical features

Patients with PART-NC predominantly present with mild cognitive impairment (Irwin et al. 2016). More pronounced cognitive impairment has been noted in another subtype of PART-NC, the so-called TPSD, where the initial symptoms usually include memory disturbances (Yamada et al. 2003). During disease progression, patients may present with disorientation, depression, and paranoid thinking, while their personality is usually well-preserved (Yamada et al. 2003; Jellinger et al. 2007). In a longitudinal analysis, increasing accumulation of neurofibrillary tangles or argyrophilic grains in PART-NC was found to be associated with faster cognitive decline (Jefferson-George et al. 2017). Comparison of longitudinal changes in cognitive performance across five domains (memory, attention, executive function, language, and visuospatial ability) and on the Mini-Mental State Examination (MMSE) showed a highly significant differences between patients with PART-NC and AD. In all these domains, AD subjects demonstrated afaster rates of decline compared to PART-NC subjects (Bell et al. 2019).

Cerebrospinal fluid (CSF) analyses in individuals with PART-NC have shown normal or elevated levels of phosphorylated tau (p-tau), like those observed in AD. Notably, a study reported that CSF p-tau181 levels rose similarly in both PART-NC and AD cases, despite the absence of amyloid plaques in PART-NC (Ericsson et al. 2023; ALZFORUM 2023).

**Fig. 5:**
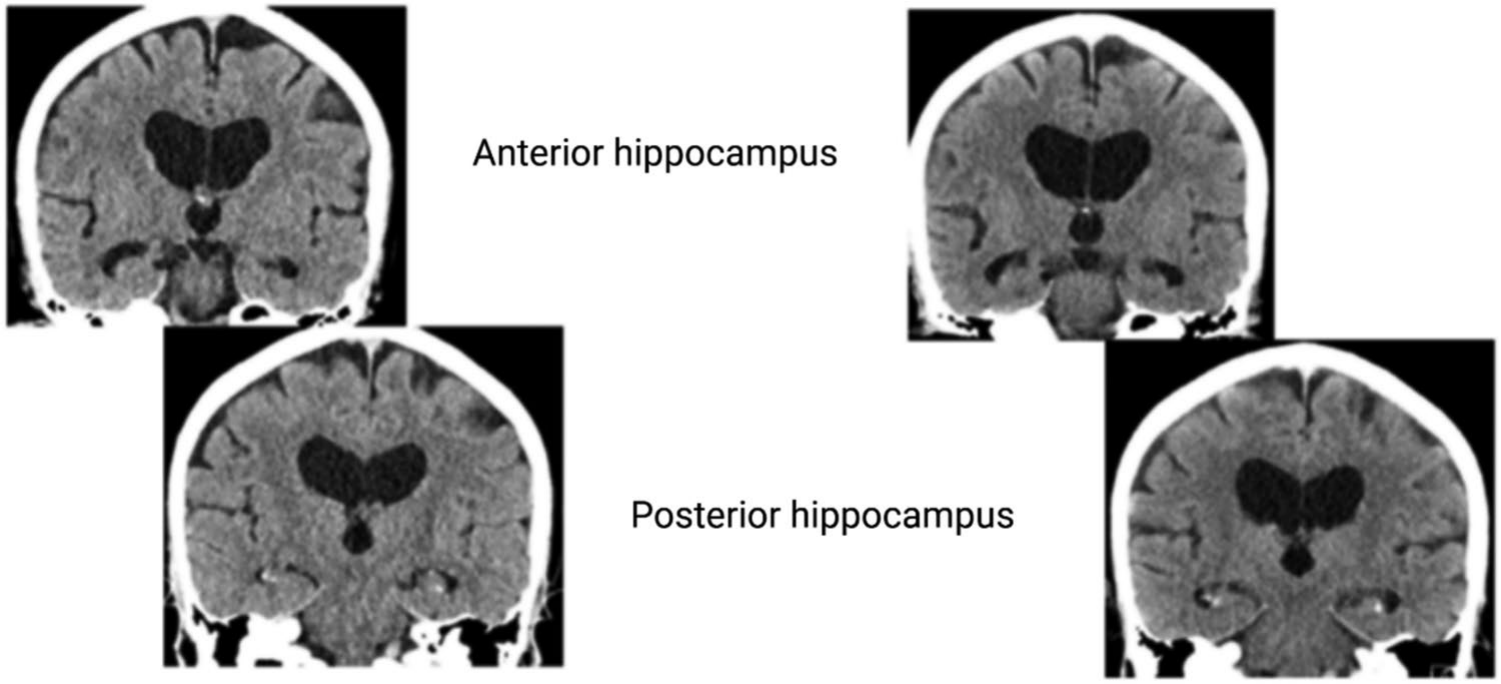
CT image of the 80-year-old patient who has an amnestic cognitive impairment accompanied by behavioral symptoms. Cognitive impairment has developed slowly over several years. Neuropsychological examination has revealed the cognitive impairment in more detail than the screening MMSE test. During the last 3 years, the atrophic changes accelerated mostly in the anterior hippocampal regions (upper row), L > R, in comparison with cortex. The patient did not have amyloid or tau pathology in the CSF and had APOE ε2/3. The clinical picture and limbic atrophy correspond more to PART. For the final diagnosis, neuropathological analysis is mandatory

**Fig. 6:**
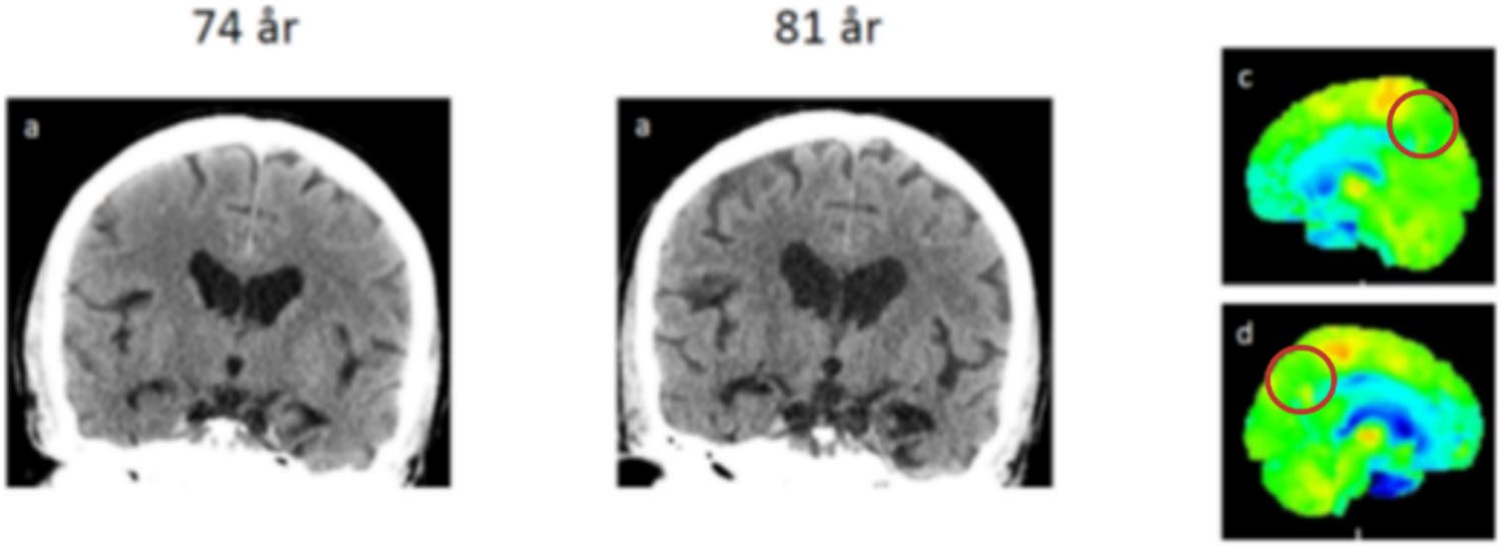
Baseline (left) and 7 years follow up CT of the brain (right) indicating accelerated atrophy of anterior hippocampus but not the cortex. [^18^F]FDG-PET showed accelerated hypometabolism (blue color) in the temporal lobe and medial frontal/cingulate cortex but not in the typical AD regions as precuneus (red ring)

Brain imaging with magnetic resonance imaging (MRI) or computed tomography (CT) can visualize differences in regional atrophy patterns between patients with PART-NC and AD. Atrophy of the anterior hippocampus, more pronounced than in the posterior hippocampus, is typically found in PART-NC subjects (Fig. 5). Atrophy of the precuneus and parietal cortex is, on the other hand, commonly associated with AD but not with PART-NC, which allows for a clear distinction in clinical practice. PART-NC also presents with a characteristic pattern of glucose hypometabolism, as evidenced by Fluorodeoxyglucose (18F) positron emission tomography ([^18^F]FDG-PET) displaying hypometabolism in the limbic areas but not in the typical AD region as precuneus (Fig. 6). Moreover, recent studies have demonstrated that tau PET imaging can identify amyloid-β-independent tau deposition in ageing individuals. For instance, research using the [^18F]RO948 tracer has shown its ability to detect tau pathology in the medial temporal lobe, aligning with regions typically affected in PART-NC (Costoya-Sánchez et al. 2023).

Subjects with PART-NC often lack the APOE ε4 allele, which is strongly associated with the increased risk of AD. The frequency of APOE ε4 in PART-NC is approximately 10%, while its prevalence in AD exceeds 45% (Frisoni 1998, Gharbi-Meliani 2021) A major genetic risk factor for PART-NC is the microtubule-associated protein tau (MAPT) gene. The MAPT H1 haplotype is a risk factor for a subtype of PART-NC, AGD, and is also an accepted risk factor for progressive supranuclear palsy (PSP) and corticobasal degeneration (CBD) (Langerscheidt 2024).

## Conclusion

Advancements in dementia diagnostics have brought us closer to identifying and understanding neuropathological conditions such as PART-NC and LATE-NC, which contribute to cognitive decline and dementia in the elderly. While clinical diagnostic criteria for LATE-NC have been established, no such formal criteria yet exist for PART-NC. Despite this, the clinical features of PART-NC closely resemble those of LATE-NC, particularly regarding age of onset and rate of progression. However, subtle but important differences are becoming apparent. For instance, LATE-NC is associated with more accelerated hippocampal atrophy than PART-NC. Moreover, CSF analysis in PART-NC cases often reveals pathological increases in phosphorylated tau, making it a potential biomarker for distinguishing PART-NC from LATE-NC.

Another key distinction lies in the cholinergic nucleus basalis of Meynert, where tau pathology is present in PART-NC, but TDP-43 pathology, typical of LATE-NC, is not. This suggests different mechanisms of cholinergic dysfunction, which could have significant implications for treatment. These differences highlight the need for tailored therapeutic approaches for PART-NC and LATE-NC.

Furthermore, discriminating LATE-NC from ADNC is crucial, particularly in the context of anti-amyloid therapies, which are currently being explored for Alzheimer’s disease (AD). Identifying LATE-NC in patients with amnestic syndromes without ADNC could lead to better personalized therapeutic strategies, preventing the misapplication of amyloid-targeting treatments in individuals who may not benefit from them. Additionally, co-pathology of ADNC with LATE-NC may significantly influence the outcomes of anti-amyloid therapeutics, further underscoring the need for precise diagnostic differentiation.

Further research is essential to clarify whether PART is a subtype of atypical AD, a part of the broader AD spectrum, or a separate clinicopathological entity. Prospective studies focusing on biomarkers, molecular imaging, and identification of distinct biological mechanisms underlying these pathologies are critical for refining their classification. Current evidence underscores the limitations of existing diagnostic tools, which remain inadequate for fully capturing the complexities of these conditions.

Addressing these diagnostic ambiguities is crucial for assigning accurate diagnoses, reducing frequent misdiagnoses of AD, and implementing appropriate therapeutic strategies for elderly patients with mild cognitive impairment or dementia. Effective communication and collaboration between clinicians, neuropathologists, and researchers are vital to refining our understanding of LATE-NC and PART-NC and the pathological, biochemical, and clinical processes they represent.

## Data Availability

The data used in this study originate from a clinical investigation involving patients at Karolinska University Hospital. Due to the sensitive nature of patient information and institutional regulations, the data are not publicly available. However, anonymized datasets may be made available from the corresponding author upon reasonable request and subject to approval by the relevant ethical review board.
